# Signs and symptoms of oral candidiasis associated with health factors and resistant *Candida* infections in a Northern Ontario patient cohort

**DOI:** 10.3389/froh.2025.1661524

**Published:** 2025-09-22

**Authors:** Karolina M. Czajka, Chris P. Verschoor, Stacey A. Santi, Danielle Brabant-Kirwan, Meredith H. Kusnierczyk, Krishnan Venkataraman, Vasu D. Appanna, Ravi Singh, Sujeenthar Tharmalingam, Deborah P. Saunders

**Affiliations:** 1 Medical Sciences Division, Northern Ontario School of Medicine University, Sudbury, ON, Canada; 2 School of Natural Sciences, Laurentian University, Sudbury, ON, Canada; 3 Health Sciences North Research Institute, Sudbury, ON, Canada; 4 Department of Medicine, McMaster University, Hamilton, ON, Canada

**Keywords:** *Nakaseomyces glabratus*, *Pichia kudriavzevii*, *Candida albicans*, antifungal resistance, oral candidiasis, older adults, pseudomembranous candidiasis, erythematous candidiasis

## Abstract

**Background:**

Oral candidiasis is a common fungal infection that disproportionately affects older adults, immunosuppressed individuals, and patients undergoing cancer treatment. Despite its prevalence, diagnosis and treatment remain challenging due to the diverse symptom presentation and potential for antifungal resistance.

**Objective:**

This study aimed to systematically evaluate which clinical signs and symptoms are most predictive of oral *Candida* infections, with a specific focus on identifying features associated with antifungal treatment failure. A secondary objective was to assess whether underlying medical conditions, including frailty and comorbidities, influence infection susceptibility or resolution following therapy.

**Methods:**

A cohort of 57 patients aged 65 years and older (mean age 74) was enrolled through oncology and hospitalist clinics in Northern Ontario. The majority (65%) were actively receiving cancer treatment. Participants underwent clinical assessment for oral candidiasis signs and symptoms, and fungal swabs were taken at baseline and two-week follow-up. Fungal species identification and treatment outcomes were recorded.

**Results:**

The majority of infections involved *Candida albicans* and responded to standard antifungal treatment. In contrast, infections involving *Nakaseomyces glabratus* and *Pichia kudriavzevii* tended to persist, consistent with known antifungal resistance. Symptomatically, pseudomembranous candidiasis—characterized by white plaques, coated tongue, and taste disturbance—was more likely to resolve, while erythematous features such as angular cheilitis and oral redness were associated with persistent infection. Although 45% of patients were classified as moderately to severely frail, frailty status was not significantly associated with infection persistence or resistance.

**Conclusion:**

These findings underscore the clinical variability of oral candidiasis and highlight the need for rapid molecular diagnostic tools at the point of care to distinguish infection types and guide appropriate therapy, particularly in older and medically complex populations.

## 1 Introduction

Oral candidiasis is an infection caused by the pathogenic overgrowth of fungal *Candida* species. Candidiasis results in local oral pain and discomfort, enhanced oral dryness, loss of taste, and aversion to food, and may lead to secondary complications such as dehydration, malnutrition, and decreased quality of life (1, 2). Furthermore, fungal infections in the oral cavity pose a risk of further consequences such as invasion into the bloodstream or to internal organ systems including the gut and digestive system, sepsis and higher mortality (3–7).

Common *Candida* species can typically live on the host's mucosal tissues in a commensal population with no pathogenicity or symptoms (8). In fact, approximately half of adults, depending on the community, have species like *Candida albicans* existing in their oral cavity without adverse consequences to their human health (8, 9). Good oral hygiene practices and dental care are an effective way to prevent fungal overgrowth and to maintain microbial homeostasis and overall good health (6, 10, 11). This includes proper dental brushing techniques, and proper care of dentures like leaving dentures out at night (12).

Numerous factors predispose an individual to developing fungal overgrowth. Infant and older adult populations, and palliative patients are highly susceptible to oral *Candida* infections, in addition to those with xerostomia (dry mouth) and disorders associated with immunosuppression and the endocrine system like HIV and cancer (2, 12–14). Factors such as polypharmacy, excessive alcohol use, lack of proper oral care, smoking, poor denture hygiene, poor nutrition and long-term steroid use or recent antibiotic and antifungal treatment can further increase susceptibility (9, 12, 15).The overall impact of different factors and how they can cause the development of infection when present in combination is still unclear.

Typical oral candidiasis symptoms in addition to dry mouth are taste disturbance and burning mouth, while angular cheilitis, removeable plaques, and oral redness are further signs. Cases can vary based on location (acute/local or systemic) and type (pseudomembranous or erythematous (9). Pseudomembranous infections which some clinicians refer to as oral thrush for acute cases, present as white lesions that resemble plaques. They can also occur chronically and become hyperplastic where the clinician cannot effectively wipe away the lesions (5). Alternatively, erythematous infections present with oral redness and burning mouth due to inflammation (16). Angular cheilitis presents with these signs at the edges of mouth with possible additional cracking (16). Accurate diagnosis requires an experienced clinician who can assess the patient's medical history for risk factors that can contribute to the development of *Candida* infection in addition to considering the range of signs and symptoms (9). Still, microbiological techniques which take time and are costly may be ultimately required to definitively identify if there is *Candida* overgrowth or an alternate condition present. This can make choosing an appropriate treatment difficult at the initial point of care as no one factor guarantees a *Candida* infection.

The main objective of this study was to determine what oral candidiasis signs and symptoms could serve as indicators for *Candida* infections especially those that are resistant to antifungal treatment. Additionally, the impact of medical conditions and frailty (physical and age-related changes that can increase the risk of illness and declining health) that may contribute to oral candidiasis and the ability to treat it successfully was assessed.

## 2 Materials and methods

### 2.1 Patients and setting

Eligible participants were identified through the North Eastern Cancer Center (NECC) Dental Clinic and Symptom Management Clinic and the Health Sciences North (HSN) Hospitalist program, all in Sudbury, Ontario, Canada. Enrollment occurred from June 2019 to September 2021. The main exclusion criterion was that individuals must not have received an antifungal treatment within two weeks prior to recruitment. The participant inclusion criteria was: age of 65 years or older currently attending the NECC Dental Oncology Clinic, Symptoms Management Clinic or HSN Hospitalist program; consent for the retrieval of two mouth swabs at the initial visit and follow-up; must be a candidate for fluconazole treatment with at least one oral candidiasis symptom present at the time of recruitment; able to attend a follow-up visit two weeks after the initial visit; and have the cognitive capacity to complete questionnaires.

Participants were surveyed to collect demographic data for their age, sex, smoking status, clinical frailty scale (CFS) (17) scores and presence of other disease comorbidities (such as high blood pressure, cardiovascular disease, diabetes, cancer etc.). Frailty scores were measured in three categories: Fit/low (CFS of 1–4), moderate (CFS of 5 or 6), severe (CFS of 7–9). Nutrition intake was assessed between normal and compromised. Oral hygiene was assessed as good, fair and poor. Smoking status was recorded as former, current or non-smoker. For dentition, patients were categorized either dentate (having natural teeth), partially dentate or edentate (no natural teeth).

### 2.2 Symptoms and fungal surveillance

The following symptoms were recorded: dry mouth, taste disturbance and burning mouth. Signs of oral candidiasis that were recorded were: angular cheilitis, coated tongue, oral redness and removable plaques (oral or oropharyngeal). An oral fluconazole treatment was prescribed as the first line of treatment for most cases displaying at least one sign or symptom. Nystatin (polyene) or Nizoral (ketoconazole) were prescribed where a topical treatment appeared more suitable depending on the location and severity of physical symptoms.

There was a follow-up after two weeks of using the prescribed antifungal treatment to assess if the infection was resolved. In cases where infections did not resolve, alternate antifungals micafungin-S (echinocandin), miconazole or clotrimazole were prescribed at the follow-up. Swab samples retrieved from the initial visit (and follow up if it persisted) were processed by the Health Sciences North (HSN) microbiology lab. Swabs were collected intraorally from the bilateral buccal mucosa and dorsum of the tongue. For fungal culturing, the samples were streaked onto Sabouraud Dextrose Agar (SDA) plates and incubated at 35°C for 48 h. Single clonal colonies were then selected for species identification using the VITEK® MS system (bioMérieux, France), a matrix-assisted laser desorption/ionization time-of-flight mass spectrometry (MALDI-TOF MS) platform. Each colony was transferred to a target slide, overlaid with a matrix solution (α-cyano-4-hydroxycinnamic acid), and allowed to dry. The samples were analyzed using the VITEK MS instrument, and species identification was determined by comparing protein mass spectral profiles against the IVD database provided by the manufacturer. Identifications with a confidence value of ≥95% were considered valid. Additionally, Glycerol stocks were created for each sample from the SDA plate colonies and stored at −80°C for future use.

### 2.3 Statistical analysis

Continuous data was summarized as the mean and standard deviation, and categorical as the count and frequency. Combinations of patient indications were presented as an upset plot, generated using the R package “ComplexHeatmap”. Associations between patient indications (dependant variable) and *Candida* species identified at baseline were estimated by logistic regression, with exception of the total indication count, which was estimated by ordered logistic regression. For the association between patient characteristics or indications and the persistence of *Candida* infection at follow-up (dependant variable), univariate estimates were calculated by logistic regression. All analyses were performed in R v4.5.0.

## 3 Results

### 3.1 Patients enrolled

**Figure 1:**
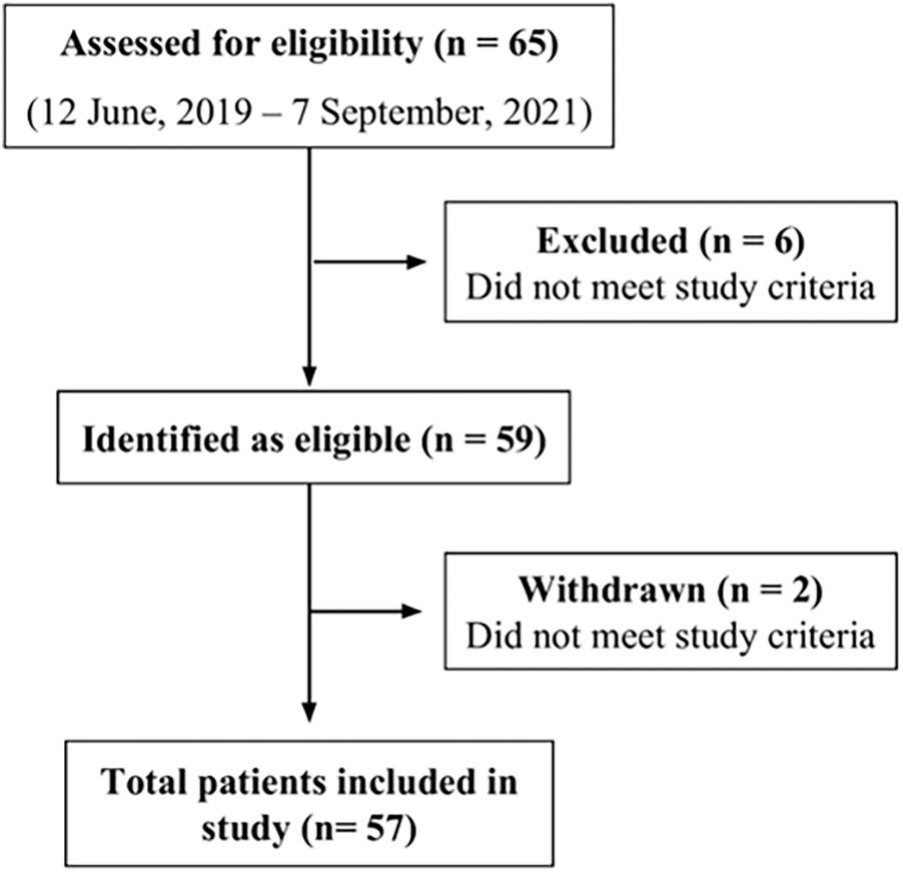
Consort chart of patient recruitment.

Of 65 patients approached for enrollment, 57 met the inclusion criterion and were enrolled in the study (Figure 1). Patients' ages ranged from 65–94 years. 33 individuals were aged between 65 and 74 years, 22 were aged between 75 and 84 years and 2 were 85 years or older. The mean age of patients participating in the study was 74 years old, with about half of patients of either sex (47% for female and 53% male) (Table 1). The majority of participants were undergoing cancer treatment (65%). Of these 37 participants, lung cancer was most common (*n* = 10) followed by tongue (*n* = 4) and myeloma, tonsil and prostate (*n* = 3 for each). Two participants each had mouth, breast or kidney cancer. There was one participant each with pelvis, bladder, skin, cheek, stomach, rectum, ear, and esophagus cancer. The cancer stage for the majority of cases was unknown (*n* = 22), followed by stage IV (*n* = 11), stage II (*n* = 3) and stage III (*n* = 1). (Supplementary Table S1) About half of participants had a frailty score of fit/low (53%), while 26% and 19% had a frailty score of mild/moderate or severe, respectively (with 1.8% missing data). For dentition status, 36% were completely dentate, 19% had partial dentition, and 24% were edentate (with 22.8% missing data). More than half (60%) had compromised nutrition intake, while for oral hygiene, most participants had fair status (61.4%) and the rest either had poor (19.3%) or good (19.3%). Only 12% of participants were current smokers, and 47% had been former smokers. Patients had individually different complex medical histories and various cardiac, vascular, pulmonary, renal, neuromuscular, liver, gastrointestinal, rheumatologic, and mental health conditions were cited among the patient cohort (Supplementary Table S2). The most common comorbid conditions were high blood pressure (*n* = 31), diabetes (*n* = 17), cardiovascular disease (*n* = 13) and gastroesophageal reflux disease (GERD) (*n* = 13).

**Table 1:** Summary of patient characteristics at baseline (*n* = 57). Continuous data summarized as the mean (standard deviation), and categorical data as the count (frequency).

Variable	Count
Age	73.8 (6.63)
Sex	
Female	27 (47.4%)
Male	30 (52.6%)
Smoking status	
Non	22 (38.6%)
Former	27 (47.4%)
Current	7 (12.3%)
Missing	1 (1.8%)
Nutritional intake	
Normal	23 (40.4%)
Compromised	34 (59.6%)
Frailty Index	0.169 (0.102)
Missing	1 (1.8%)
Clinical Frailty Score	
Fit/Low	30 (52.6%)
Mild/Moderate	15 (26.3%)
Severe	11 (19.3%)
Missing	1 (1.8%)
Undergoing cancer treatment	
No	20 (35.1%)
Yes	37 (64.9%)
Oral hygiene status	
Poor	11 (19.3%)
Fair	35 (61.4%)
Good	11 (19.3%)
Dentition status	
Dentate	21 (36.8%)
Partially Dentate	11 (19.3%)
Edentate	12 (21.1%)
Missing	13 (22.8%)

### 3.2 Baseline fungal screening and symptomology

**Figure 2:**
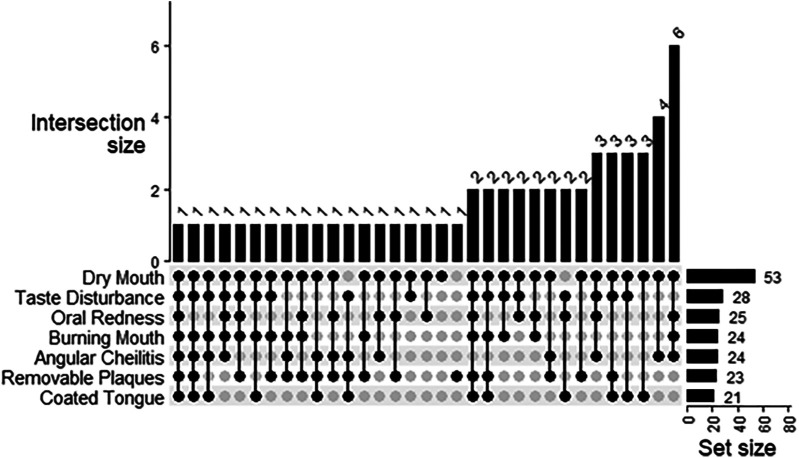
Combinations of presenting indications in patients at baseline.

**Figure 3:**
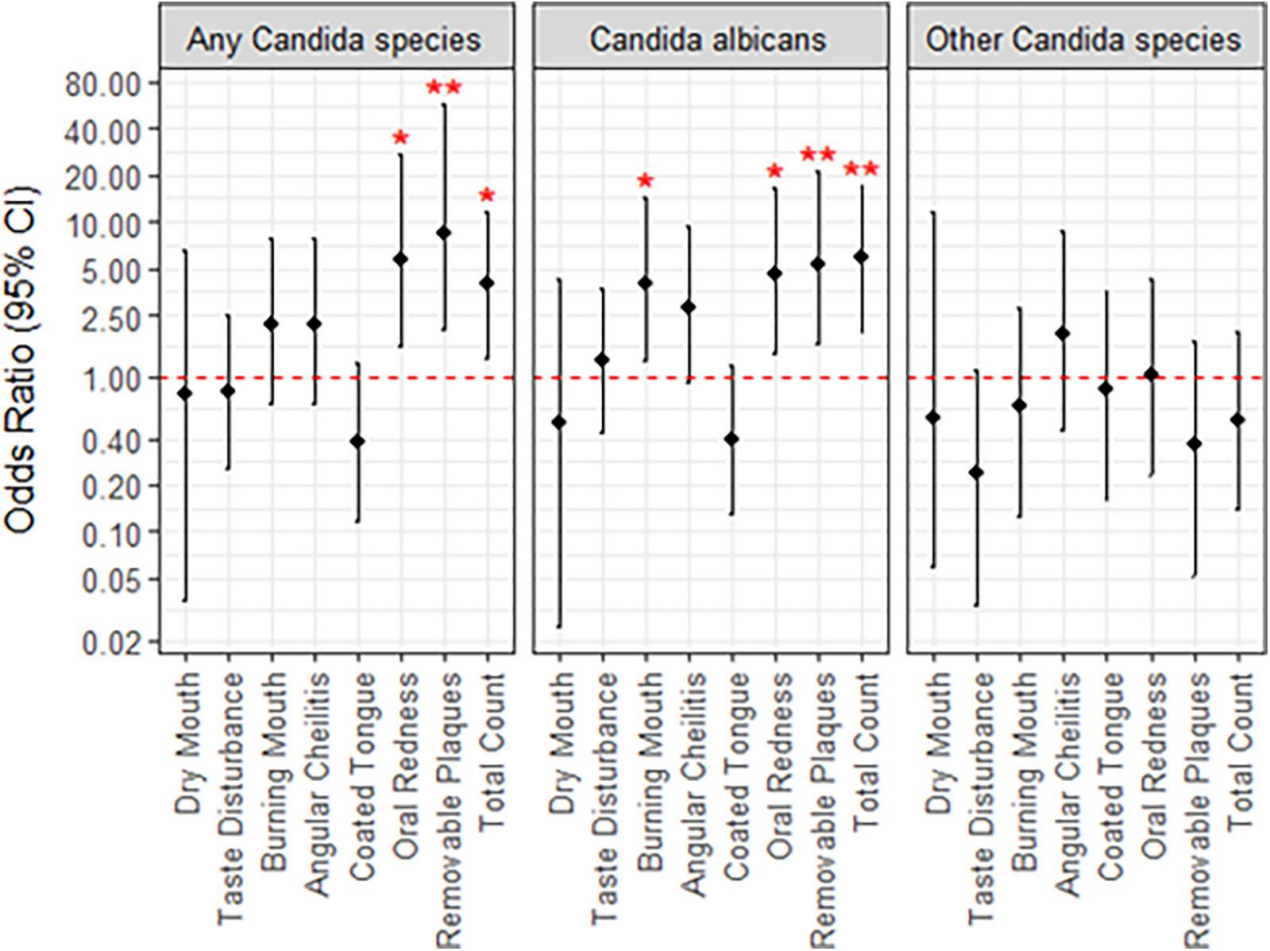
Univariate associations between *Candida* species detected at baseline and patient indications. Lack of significance is indicated by overlap with the red, dotted line, and asterisks; **, *p* < 0.01; *, *p* < 0.05.

Almost all the participants presented with the dry mouth symptom (93.0%) (Figure 2). About half of participants experienced taste disturbance (50.9%), while about 40% of patients had burning mouth (42.1%), angular cheilitis (42.1%), oral redness (43.9%) or removable plaques (40.4%). A coated tongue was present least frequently (36.8%). Only one participant presented with all six symptoms of oral candidiasis (Figure 2). The most frequent combination of symptoms was dry mouth, angular cheilitis, redness and burning (*n* = 6). Using logistic regression analysis, the odds of experiencing redness [OR (95% CI) = 5.7 (1.57, 27.6)] or removeable plaques [8.3 (2.0, 57.1)] was significantly higher with the presence of any *Candida* species infection (Figure 3). The total number of indications present was also significantly associated with the presence of any Candida species, where the odds of an increased indication count is 3.9-times (95% CI = 1.32, 11.8) higher. There was a similar result if *Candida albicans* was specifically present at baseline [oral redness: OR (95% CI) = 4.5 (1.44, 16.4); removable plaques: 5.3 (1.61, 21.5); and total indication count: 5.9 (1.98, 17.4)] in addition to significantly increased odds of having burning mouth [4.0 (1.28, 14.6)] (Figure 3). No significant associations were observed between patient indications and species other than *Candida albicans*.

Most infections (57.6%) were treated with the first-line antifungal agent fluconazole. Nystatin or Nizoral was prescribed in 32.3% of cases as alternatives, typically based on infection severity and anatomical location within the oral cavity. In total, approximately 65 clinical isolates were retrieved from the initial visit (*n* = 48) and follow-ups (*n* = 15). From the baseline swab samples, 17 (29.8%) were identified as normal flora since the concentration of *Candida* did not surpass the pathological threshold (Table 2). The majority of abnormal flora samples contained *Candida albicans* (61.0%). The second most common species was *Nakaseomyces glabratus* (*n* = 6,) and there were a few other relevant species: *Pichia kudriavzevii* (*n* = 1), *Candida tropicalis* (*n* = 2), and *Candida lusitanae* (*n* = 1) (Table 2). Eight infections at the initial visit and four infections at the follow-up contained more than one fungal species (Table 3).

**Table 2:** Summary of antifungal treatment and detected species at baseline. *Candida* species present in oral candidiasis samples.

Variable	Total
	(*N* = 57)
Antifungal prescribed	
Fluconazole	34 (59.6%)
Nystatin	18 (31.6%)
Nizoral	4 (7.0%)
Missing	1 (1.8%)
Normal flora	
N	40 (70.2%)
Y	17 (29.8%)
*C. albicans*	
N	22 (38.6%)
Y	35 (61.4%)
*C. glabrata*	
N	51 (89.5%)
Y	6 (10.5%)
*C. krusei*	
N	56 (98.2%)
Y	1 (1.8%)
*C. lusitaniae*	
N	56 (98.2%)
Y	1 (1.8%)
*C. tropicalis*	
N	55 (96.5%)
Y	2 (3.5%)

**Table 3:** Presence of two yeast species (*Candida* and others) in 9 patients.

Species	Initial Visit (Absolute Numbers)	Follow-up (Absolute Numbers)
*Candida albicans* + *Candida glabrata*	4	3
*Candida albicans* + *Candida tropicalis*	2	0
*Candida albicans* + *Candida parapsilosis*	0	1
*Candida albicans* + *Rhodotorula mucilaginosa*	1	0
*Candida krusei* + *Sachharomyces cerevisiae*	1	0

### 3.3 Follow-up outcomes and regression analysis

**Figure 4:**
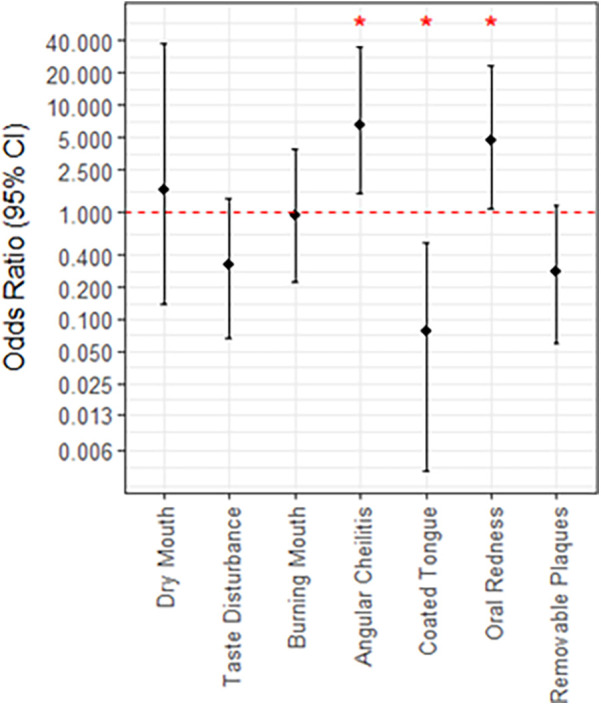
Associations between patient indications at baseline and the persistence of *Candida* infection at follow up. Lack of significance is indicated by overlap with the red, dotted line, and asterisks; *, *p* < 0.05.

After two weeks, many infections were successfully treated and no remaining *Candida* was detected, thus there was no follow-up sample collected. Additionally, 12 patients were deceased before the follow-up or otherwise did not attend. After the follow-up was completed, 15 patients (26.3%) had a persistent *Candida* infection (5 *C. albicans, 2 N. glabratus,* 3 *C. albicans* *+* *N. glabratus*, 2 *C. parapsilosis,* 1 *C. albicans* *+* *C. parapsilosis,* 1 *P. kudriavzevii* and 1 *C. tropicalis)* while it was resolved in 18 patients (31.6%). If the patient presented with oral redness [OR (95% CI) = 4.68 (1.09, 22.9)] or angular cheilitis [6.50 (1.47, 34.3)]) at the initial visit, there were significantly increased odds that the infection would be unresolved at the two-week follow up (Figure 4). Alternately, patients presenting with coated tongue were less likely to have their *Candida* infection resolved by the time of the follow-up [0.08 (0.01, 0.51)](Figure 4). In terms of the *Candida* species present for unresolved infections at follow up, there was no significant association with *C. albicans*, *N. glabratus*, or other *Candida* species. Patients with a confirmed *N. glabratus* infection at baseline were significantly more likely to be infected at follow-up [OR = 9.4 (1.27, 196.2)], although confidence intervals were particularly wide. Of the other patient factors considered, such as age, smoking status and antifungal prescribed, only sex was found to be significantly associated to resolution of infection at follow-up (Supplementary Figure S1). Specifically, males were found to be less likely than females [0.2 (0.04, 0.86)]. Univariate estimates for the indications oral redness, angular cheilitis and coated tongue were slightly reduced after adjusting for male sex, although all retained significance at *p* < 0.20.

## 4 Discussion

Overall, this study aimed to identify which signs and symptoms of oral candidiasis in addition to what risk factors can aid in correctly diagnosing a *Candida* infection and can predict whether the prescribed treatment will be successful. We found that a variety of risk factors may be present, and no single factor was significantly associated with the presence of a *Candida* infection. Alternately, the presence of oral redness or removeable plaques did increase the likelihood that there was *Candida* overgrowth and this was further increased with a higher total count of signs and symptoms present. There was a similar result for *Candida albicans* specifically, except burning mouth also increased the likelihood. At the follow-up after a two-week treatment, those who initially presented with oral redness or angular cheilitis had significantly increased odds that infection would not be resolved. In comparison, the presence of a coated tongue suggested increased odds that the infection would be cleared up.

### 4.1 Signs and symptoms of oral candidiasis

The results indicate that *Candida* infections do not all present uniformly as demonstrated by the varying combinations of oral candidiasis signs and symptoms. Removeable plaques are one of the most salient indicators in diagnosing this infection, however our results indicate the importance of considering all possible features. Diagnosis of pathogenic *Candida* strains could be missed since almost all patients had dry mouth and plaques cannot form without adequate saliva. In fact, the most frequent combinations of symptoms (dry mouth, angular cheilitis, oral redness and burning mouth) did not include plaques. These appear to be just as relevant for accurate diagnosis as indicated by the strong statistical association with the presence of plaques, oral redness and the total count of signs and symptoms with increased odds of a *Candida* infection.

Dry mouth was the most ubiquitous of the symptoms that were assessed as it was experienced by 90% of our patient population. The lack of variable variance likely resulted in no statistical significance when considering chances of having a *Candida* infection. Nonetheless, this symptom calls for closer examination as there was no case of oral candidiasis without this feature. A study by Alt-Epping (*et al*., 2012), had a similar finding, where almost 90% of their patient cohort also had dry mouth. Their cohort had a higher frequency of taste disturbance as well (∼80%) in comparison to the approximate 50% of our study (18). The symptom of xerostomia can have a negative consequence where those lacking adequate saliva cannot benefit from the natural and protective antifungal proteins like histatins, defensins and lysozymes secreted in saliva (10). Targeting treatment to restore salivary gland functioning could aid in prevention of future infection (18).

### 4.2 Factors associated with increased risk of oral candidiasis

Oral *Candida* infections can occur with equal frequency in both males and females as our patient survey data demonstrates (9). In terms of other health factors like frailty scores and oral hygiene, there were no statistically significant predictors of a persistent *Candida* infection. This highlights that many older adult patients susceptible to pathogenic *Candida* infections will have a complex medical history and the range of health factors needs to be considered on a case-by-case basis.

Frailty is a complex, age-related syndrome of enhanced vulnerability to a myriad of health outcomes and has been described as the “most problematic expression of aging” (19). Elevated frailty has been linked to microbial dysbiosis, oral disease and infection, emphasizing its important role in driving disease risk beyond what can be attributable to age (20, 21). This is a bidirectional relationship, as poor oral health such as loss of natural teeth also exacerbates frailty, leading to worse health outcomes including earlier mortality (22). Frailty was not significantly associated with infection persistence or resistance in the 45% of patients in our study classified as moderately to severely frail. The limited size of the patient cohort may attribute to the lack of significance that was determined.

Diabetes is an additional condition that is associated with a higher prevalence of oral candidiasis and oral symptoms like dry mouth (23). Higher concentrations of glucose in saliva and blood can enable the pathogenic switch of *Candida spp.* that can turn into an invasive infection (24). One study found in diabetes patients that *Candida* species have higher enzymatic activity (hemolytic and phospholipases) which can break down membranes and invade epithelial tissues (24). In our cohort, 18 of the 57 patients had diabetes, which could increase their susceptibility to chronic and invasive infection.

### 4.3 Follow-up outcomes

The sign of white plaques trended towards being cleared up by the follow-up exhibiting that pseudomembranous infections can be easier to adequately treat. Coated tongue and taste disturbance symptoms also trended similarly which correlates well with the overall presentation of this infection type. In this case, the topical antifungals are quite effective. The odds of having no *Candida* infection at the follow-up was higher with a coated tongue present possibly because this symptom can occur due to other reasons like poor oral hygiene or xerostomia (25–27).

Alternatively, erythematous candidiasis with symptoms like angular cheilitis can be more difficult to treat and can occur chronically (28). The significant odds of persistent *Candida* infection for this symptom and redness are understandably correlated as one of the key features of angular cheilitis is the redness and cracking at the edges of the mouth. In these cases, the clinician may not accurately diagnose the presence of oral candidiasis and topical antifungals are not sufficient to fully resolve the infection. Prescribing a systemic antifungal would be a better choice for *C. albicans* strains. If the infection contains intrinsically resistant species like *N. glabratus* or *P. kudriavzevii,* then an alternate prescription is required such as an echinocandin. *Nakaseomyces glabratus* and *Pichia kudriavzevii* have been reclassified taxonomically, but they have still been included in this analysis alongside other *Candida* species for consistency with historical literature. Both species are clinically relevant as fungal pathogens that cause oral candidiasis.

There was no significant correlation between the type of *Candida* species and whether the infection was resolved at the two-week follow-up. This corroborates that most *C. albicans* are susceptible to antifungals and do not have innate resistance to the azole or polyene drug class (2). For *N. glabratus*, there were 5 out of 6 total *N. glabratus* infections still present at the follow-up, likely due to its intrinsic azole resistance. Notably, *Candida parapsilosis* presented in three follow up infections and none were identified at baseline. This suggests a mycobiome shift during antifungal treatment that enabled this species to colonize the infection over *C. albicans*. Recent studies indicate the increasing emergence of fluconazole-resistant *C. parapsilosis* strains when previously this species was typically susceptible to first-line treatment (29, 30).

One of the cleared infections with initial *C. albicans* and *N. glabratus* strains instead presented with another fluconazole-resistant species *P. kudriavzevii* at the follow up. Additionally, some patients can have numerous pathological *Candida* species comprising the infection, which further emphasizes the usefulness of a sensitive and quick point-of-care species identification to prescribe antifungal treatment that can treat the entire infection.

### 4.4 Conclusions and future directions

This study's featured strengths include real world setting, longitudinal design and comprehensive characterization of patients and clinical *Candida* samples. The data contributes to filling in the gaps of knowledge specifically for Northern Ontario cohorts. Limitations include the sample size and wide range of medical history which made it more difficult to ascertain health factors as indicators for oral candidiasis Adequate sample numbers in future studies could enable segregation of participants based on cancer types or other immunocompromising conditions. The study's applications are also limited by geographic location, as it is well known that the prevalence of different *Candida* species can vary based on this factor. For both Canada and the United States, *Candida albicans* is usually cited as the most common pathogenic species followed by *Nakaseomyces glabratus* (31–33)*.* Furthermore, the limited number of persistent cases at follow-up reduced the statistical power of the analysis. Again, higher sample numbers could better indicate if there are clinical signs and symptoms that significantly correlate with less common species that display more antifungal resistance. This is of particular importance considering the rising incidence of uncommon *Candida* spp. and its impact on higher mortality (34). Also, numerous patients exhibiting signs and symptoms of infection had only normal flora identified. Indeed, numerous disorders like Sjorgen disease can present with similar features that are not directly a result of *Candida* overgrowth (35). Future studies should consider a more holistic approach for studying the oral microbiome which may reveal what other organisms are involved in maintaining normal floral homeostasis.

In summary, our study emphasizes the variation in presentation of oral candidiasis and the need for molecular diagnostic tools that can aid the physician in accurate diagnosis. Physicians need to consider a host of factors when treating patients showing signs or symptoms to prescribe the appropriate antifungal. Furthermore, there are *Candida* species with innate resistance to first line agents as well as strains with acquired resistant mutations that are difficult to pre-emptively identify without microbiological culturing and testing (2). Current methods for identifying oral candidiasis require a skilled lab technician to culture strains from a swab and then using the VITEK® assay which identifies fungal species based on a mass-spectrometry protein profile (2).

When patients presented with *Candida albicans* as the primary infection, the standard fluconazole treatment typically worked sufficiently. Erythematous infections with symptoms like angular cheilitis and oral redness trended towards persisting, especially when treated with topical agents like nystatin or Nizoral. A systemic treatment of fluconazole may have been more effective. However, intrinsically resistant species like *Nakaseomyces glabratus* (formerly known as *Candida glabrata*) were not effectively treated with fluconazole. In fact, 83% of *Nakaseomyces glabratus* infections (5/6) persisted at the follow-up and required an alternate antifungal treatment to be prescribed. The clinical indicators of oral candidiasis cannot distinguish between these various species and molecular species characterization would be required to identify antifungal-resistant species.

The development of a point-of-care species identification could enable clinicians to more accurately assess *Candida* infections and prescribe the appropriate treatment. Other species that are resistant to fluconazole include *Pichia kudrivzevii* (*formerly known as Candida krusei*) and the recently emerged multi-drug resistant *Candida auris.* More than 95% of *P. kudriavzevii* isolates and about 90% of *C. auris* strains are typically resistant to fluconazole (36, 37). Though *Candida auris* has not yet been identified in the oral cavity of a human patient, it was retrieved from the oral cavity of a dog in Kansas (38). Being able to accurately identify this species along with the more expected *Candida* species would be helpful for monitoring the potential spread and evolving modes of transmission (39).

There are few in-depth analyses that consider the variety of oral candidiasis signs and symptoms, patient health survey data, and microbiological testing of samples. Future studies will include susceptibility testing, and molecular gene expression data for the samples collected with a focus on resistant strains. This combination of analyses will help to elucidate the pathophysiology of oral candidiasis infections in susceptible populations like immunocompromised and older adults.

## Data Availability

The original contributions presented in the study are included in the article/Supplementary Material, further inquiries can be directed to the corresponding author.
