# Supplementary material for: Signs and symptoms of oral candidiasis associated with health factors and resistant Candida infections in a Northern Ontario patient cohort
Source: Front Oral Health. 2025 Sep 22;6:1661524. doi: 10.3389/froh.2025.1661524 (PMC12498526; doi:10.3389/froh.2025.1661524)

Supplementary Materials

**Table S1:** Range of cancer types and cancer stages present in the patient cohort.

| **Cancer Type** | **Number of patients** |
| --- | --- |
| NA | 20 |
| Lung | 10 |
| Tongue | 4 |
| Myeloma | 3 |
| Tonsil | 3 |
| Prostate | 3 |
| Mouth | 2 |
| Breast | 2 |
| Kidney | 2 |
| Pelvis | 1 |
| Bladder | 1 |
| Skin(Canthus) | 1 |
| Cheek | 1 |
| Stomach | 1 |
| Rectum | 1 |
| Ear | 1 |
| Esophagus | 1 |
| Total | 57 |
|  |  |
| **Cancer Stage** | **Number of patients** |
| Unknown | 22 |
| IV | 11 |
| II | 3 |
| III | 1 |

**Table S2:** Range of different health factors recorded for the patient cohort.

| **Medical History** | **Number of Patients** |
| --- | --- |
| High blood pressure | 31 |
| Diabetes | 17 |
| Cardiovascular disease | 13 |
| GERD | 13 |
| Chest/Lung | 10 |
| High cholesterol | 9 |
| Arthritis | 8 |
| Asthma | 8 |
| Osteoporosis | 6 |
| Palliative | 4 |
| Depression | 4 |
| Anemia | 4 |
| Ear/Sinus | 4 |
| Hypothyroidism | 3 |
| Anxiety | 3 |
| Dyslipidemia | 3 |
| Alcoholism | 3 |
| Cataracts | 2 |
| Renal Disease/ Failure | 2 |
| COPD | 2 |
| Blood Clots | 2 |
| Fever/Infection | 1 |
| Neuro | 1 |
| Headaches/migraines | 1 |
| Osteoarthritis | 1 |
| Bipolar | 1 |
| Parkinsons | 1 |
| Diverticulitis | 1 |
| Sleep Apnea | 1 |
| Cervical Stenosis | 1 |
| Carpal Tunnel | 1 |
| Hepatitis | 1 |
| Autoimmune disorder | 1 |

**Figure S1:** Odds ratio of any health factors and other study variables being associated with an antifungal resistant *Candida* infection. Lack of significance is indicated by overlap with the red, dotted line, and asterisks; *, p<0.05.


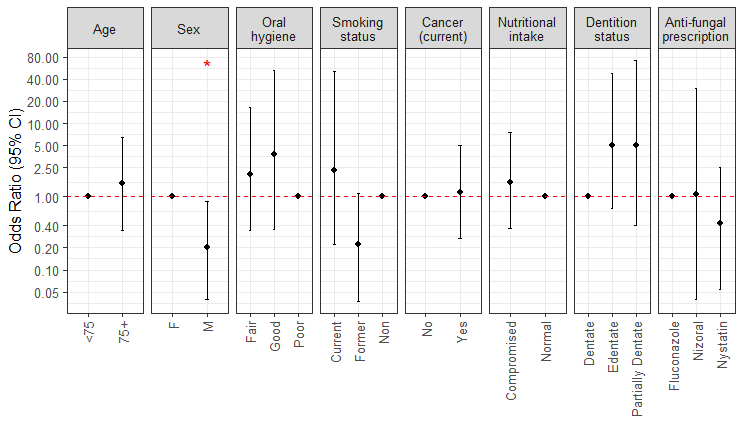

Supplement: Supplementary file 1 [file Table1.docx]
